# A QTL approach in faba bean highlights the conservation of genetic control of frost tolerance among legume species

**DOI:** 10.3389/fpls.2022.970865

**Published:** 2022-10-19

**Authors:** Estefanía Carrillo-Perdomo, Jean-Bernard Magnin-Robert, Blandine Raffiot, Chrystel Deulvot, Matthieu Floriot, Isabelle Lejeune-Hénaut, Pascal Marget, Judith Burstin, Nadim Tayeh, Grégoire Aubert

**Affiliations:** ^1^ Agroécologie, INRAE, Institut Agro, Univ. Bourgogne, Univ. Bourgogne Franche-Comté, Dijon, France; ^2^ UMR AGAP Institut, Univ. Montpellier, CIRAD, INRAE, Institut Agro, San Giuliano, France; ^3^ Terres Inovia, Thiverval-Grignon, France; ^4^ Agri Obtentions, Ferme de Gauvilliers, Orsonville, France; ^5^ Département de génétique et protection des cultures, BioEcoAgro Joint Research Unit, INRAE, Université de Lille, Université de Liège, Université de Picardie Jules Verne, Estrées-Mons, France; ^6^ INRAE, UE115 Domaine Expérimental d’Epoisses, Dijon, France

**Keywords:** Frost tolerance, faba bean (Vicia faba L.), genetic linkage mapping, recombinant inbred line (RIL) population, quantitative trait loci (QTL), synteny, barrel medic (Medicago truncatula G.), pea (Pisum sativum L.)

## Abstract

Frost is a major abiotic stress of winter type faba beans (*Vica faba* L.) and has adverse effects on crop yield. Climate change, far from reducing the incidence of frost events, is making these phenomena more and more common, severe, and prolonged. Despite the important interaction that the environment has in the tolerance of faba bean to frost, this trait seems to have good levels of heritability. Several QTLs for frost tolerance have already been reported, however, a more robust identification is needed to more precisely identify the genomic regions involved in faba bean tolerance to sub-zero temperatures. Several pea (*Pisum sativum* L.) and barrel medic (*Medicago truncatula* L.) frost tolerance QTLs appear to be conserved between these two species, furthering the hypothesis that the genetic control of frost tolerance in legume species might be more generally conserved. In this work, the QTL mapping in two faba bean recombinant inbred line (RIL) populations connected by a common winter-type parent has led to the identification of five genomic regions involved in the control of frost tolerance on linkage groups I, III, IV, and V. Among them, a major and robust QTL of great interest for marker-assisted selection was identified on the lower part of the long-arm of LGI. The synteny between the faba bean frost tolerance QTLs and those previously identified in other legume species such as barrel medic, pea or soybean highlighted at least partial conservation of the genetic control of frost tolerance among different faba bean genetic pools and legume species. Four novel RILs showing high and stable levels of tolerance and the ability to recover from freezing temperatures by accumulating frost tolerance QTLs are now available for breeding programs.

## Introduction

Faba bean (*Vicia faba* L.) is a cool season rain-fed legume and as such a key component of sustainable agriculture and food security due to its high nutritional value and the ecological services it provides. Faba bean seeds are rich in proteins, carbohydrates, dietary fibers, and micronutrients (Mulualem et al., 2012). They are important components of human food and animal feed in many countries. The crop improves soil fertility through symbiosis with nitrogen-fixing bacteria and is capable of increasing the yield of other crops such as cereals when included in rotations (Duc, 1997; Crépon et al., 2010; Maqbool et al., 2010). Faba bean cultivars are divided into spring or winter types. Their sowing season (spring/autumn/winter) varies according to the climate including the risk of frost in the cropping region (Flores et al., 2013). In this sense, there are three cultivation arrangements: i) in the Mediterranean areas, cultivars should be adapted to autumn sowing; (ii) in oceanic or fairly mild-winter continental climate areas, cultivars should be of winter-type and adapted to autumn sowing and; (iii) in cold regions of northern or continental Europe, cultivars should be of spring-type, adapted to early-spring sowing (Annicchiarico et al., 2015). When sown in autumn or early winter, the crop has to withstand low temperatures that can freeze its aerial organs and its roots, from the early stages of its development.

Frost is a major abiotic stress for winter-type faba beans and has adverse effects on crop production (Sallam et al., 2016a; Sallam et al., 2016b). The combination of climatic factors such as the rate of temperature decrease, the intensity and the duration of the freezing period, the intensity of the wind, the daily insulation, and the absence/presence of snow cover are determinants in the survival of faba bean plants (Duc et al., 2011). Low temperatures threaten plants by inducing the formation of ice crystals in plant tissues (Ambroise et al., 2020) and causing dehydration (Ambroise et al., 2020). Frost-damaged faba bean plants have leaves with a water-soaked appearance that wilt and turn dark brown or black. Although frost damage is less common on the roots thanks to soil insulation, below-ground freezing temperatures can seriously impact both the thin roots that absorb water and minerals and the symbiosis with nitrogen-fixing bacteria, thus affecting nitrogen fixation (Stoddard et al., 2006; Ambroise et al., 2020). After a frost event plants still have to de-acclimate to survive thawing. The thawing rate plays a key role in survival chances since water must be absorbed faster than it is released (Scarth, 1944). Particularly harmful are late frost events that occur once plants have already begun to mobilize their reserves to resume sprouting or have started the reproduction phase (Maqbool et al., 2010; Duc et al., 2011). Late frosts can cause the abortion of buds and flowers and affect pod formation and seed filling (Duc et al., 2011). In addition, frost damages make the plants more vulnerable to pests and pathogens (Maqbool et al., 2010).

However, cold acclimation occurs naturally in faba bean fields during the course of fall to winter. As described in other plants (Thomashow, 2010; Guy, 2011; Liu et al., 2019), faba bean has the capacity to improve its response to freezing after going through a cold acclimation period. During this period, the plants are exposed to successive days of progressively decreasing positive temperatures. This is usually correlated with an increasingly shorter photoperiod. During cold acclimation, changes in gene expression occur that lead to biochemical and physiological adaptations reinforcing the plant’s ability to subsequently cope with intense frosts.

Frost tolerant faba bean varieties can survive frost or freezing temperatures. However, surviving plants (tolerant plants) need to invest time and resources to recover and regenerate. It is achieved at the cost of compromising their potential productivity. Different strategies can be activated simultaneously by the plant to tolerate and survive face frost: (i) limitation of the formation of ice crystals and prevention of freezing if water is kept liquid below 0°C (supercooling), (ii) insulation of cells from low temperatures by modifying the lipid bilayer of the cell wall and; (iii) tolerance to the presence of ice crystals and dehydration (Ambroise et al., 2020).

The warming caused by climate change, far from reducing the frequency, severity and duration of frost events, is making these phenomena increasingly problematic and common in many regions of the world (Gu et al., 2008). The less extreme temperatures that are being reached in winter, destabilize the climatic conditions of the poles and cause an increase in extreme meteorological events such as early and late frosts (Francis and Skific, 2015). Milder autumn temperatures could affect the efficiency of faba bean cold acclimation, as is the case for pea, another grain legume species (Castel et al, 2017). Furthermore, early and late frosts affect plants during the most critical stages of their life cycles (seedling, sprouting and, reproduction), making the development of frost-tolerant varieties strategic to ensure the future of the crop.

Within legumes, the genetic determinism of frost tolerance has been studied in the model plant *Medicago truncatula* (Avia et al., 2013; Tayeh et al., 2013a), in the forage crop *Medicago sativa* (Brouwer et al., 2000; Li et al., 2015; Adhikari et al., 2018, 2021) and in important grain legume crops such as pea (Dumont et al., 2009; Zhang et al., 2011; Klein et al., 2014), faba bean (Arbaoui et al., 2008; Ali et al., 2016; Sallam et al., 2016a; Sallam et al., 2016b), lentil (Kahraman et al., 2015) and chickpea (Kahraman et al., 2004). In all these species, the trait appears to be polygenic and some results have highlighted syntenic QTLs between pea (Avia et al., 2013; Tayeh et al., 2013a) and *M. truncatula* and between faba bean and *M. truncatula* (Ali et al., 2016). In faba bean, several cultivars and breeding lines capable of withstanding sub-zero temperatures have been identified (Stoddard et al., 2006; Arbaoui et al., 2008b; Duc et al., 2011; Annicchiarico et al., 2015). Despite important environmental effects on frost tolerance (Duc et al., 2011; Flores et al., 2013; Sallam et al., 2016a), this trait appears highly heritable and polygenic, with large additive effects (Duc and Petitjean, 1995). Several quantitative trait loci (QTL) related to frost tolerance, winter hardiness, fatty acid content (FAC), and yield have been reported for this species (Arbaoui and Link, 2008; Arbaoui et al., 2008a; Sallam et al., 2015; Sallam et al., 2016b). Although several QTLs have already been identified, it would be necessary to carry out QTL analyses on more saturated genetic linkage maps that allow reducing the confidence interval of the QTLs to facilitate the identification of molecular markers useful in marker-assisted selection (MAS).

Considering that the faba bean genome sequence was not available at the time this study was conducted, the strategy to identify QTLs for frost tolerance in faba bean was to perform QTL mapping on two bi-parental populations with diverse genetic backgrounds. We identified genomic regions that control frost tolerance in a field trial network, using two faba bean recombinant inbred line (RIL) populations connected by a common winter-type parent. We used the densest faba bean consensus map (Carrillo-Perdomo et al., 2020) to date to study the synteny between the frost tolerance QTLs identified in faba bean and those previously identified in other legume species to highlight genomic regions useful for marker-assisted selection.

## Materials and methods

### Plant material

Two populations of connected recombinant inbred line (RIL) families *POP2* and *POP3* described by Carrillo-Perdomo et al. (2020) have been used in this study (**Figure S1**). Both populations share Hiverna (IVIP102372, accession code of the INRAE faba bean collection) as a common female parent, and Silian (IVIP100340) and Quasar (IVIP102378) are the respective male parents for *POP2* and *POP3*. Cultivar Hiverna (Germany) is known to be well adapted to continental climates (Link et al., 2010) while Quasar (United Kingdom) is a winter cultivar bred in England (Ondrej and Hunady, 2007) and thus adapted to oceanic climates (cool winters with abundant rainfall). Silian is a *V. faba* spp. *minor* landrace from Northern Sudan susceptible to frost. RILs used for phenotyping were F_5:6_ families produced by single seed descent (SSD) until the F3 generation. *POP2* and *POP3* consisted of 145 and 96 RIL families respectively. Genotyping data and genetic maps described by Carrillo-Perdomo et al. (2020) were used for further QTL analyses.

### Multi-year and multi-location field experiments: Frost damage assessments

Three experiments were carried out: i) a first trial *(B_2016-17*), at the Epoisses experimental farm of INRAE in Bretenière (France) (Latitude 47° 23 ‘70 ′ ′ N; Longitude 5° 09 ′ 80 ′ ′ E; Altitude 210 m), was sown on November 3, 2016; ii) a second trial *(B_2017-18*), at the Epoisses experimental farm, was sown on October 16, 2017; and iii) a third trial (*O_2017-18*), in Orsonville (France) (Latitude 48° 47 ‘77’ ‘N, 1° 83’ 53 ‘‘ E) was sown on November 3, 2017. Climatic parameters recorded at nearby weather stations are available in **Table S1** and **Figure S1** for each environment. Each trial consisted of a complete randomized two-block design. For each RIL and parent of the two populations, twenty seeds were sown in rows of 2.5 m with a 1 m distance between rows. The trials were chemically protected against weeds, fungi, and pests, when necessary. Freezing events were defined as periods of at least five successive days with negative temperatures measured under shelter. Frost damage (*FD*) was scored 15 to 30 days after freezing events. Therefore, the number of evaluations depended on the number of frost events that occurred at each location. A scale of 0 to 5 (**Figure S2**) was used to evaluate the symptoms of frost damage (*FD*) in plants: zero corresponded to no visual damage and five corresponded to dead plant. The area under the symptoms progress curve (*AUSPC*) (Wilcoxson et al., 1975) was calculated in each environment when more than one evaluation of frost damage was available to evaluate its progress over time. The equation used was: $\sum_{i=1}^{n-1}\frac{y_{i}\;+\;y_{i+1}}{2}\;x\;(t_{i+1}-\;t_{i})$ ; where *y_i_* is the assessment of frost damage at the *i*th observation, *t_i_* is time in days at the *i*th observation, and *n* is the total number of observations. Additionally, the number of plants per accession and block was counted from the emergence to the end of the winter season. Survival rate (*SR*) was assessed once at the end of the winter season by counting the percentage of surviving plants for each accession.

### Statistical analysis

Analysis of variance (ANOVA) for a complete block randomized design was conducted to test significant differences among accessions and environments for *FD*, *AUSPC*, and *SR* with accession (G) and environment (E) as fixed factors in order to determine the genotypic (G) and the genotype × environment (G×E) interaction effects for frost tolerance in *POP2* and *POP3*. We used the statistical model: y_ijkl_ = μ + E_i_ +R(E)_j(i)_ + G_k_ + GE_jk_ + ϵ_ijkl_ where, i = 1,…,145 for *POP2* and i = 1,…,96 for *POP3*; j = 1,…,3; k = 1, 2; and yijk denotes the response variable of genotype i, in environment j, block k; μ is the mean of all plots; E_i_ is the environmental main effect of trial i; R(E)_j(i)_ stands for the effect of the block j in trial l; G_k_ is the genetic effect of genotype k; GE_jk_ is the genotype-by-environment interaction effect for genotype k in trial i, and ϵ_ijkl_ is the plot residual term. The underlined terms were considered random effects, which were assumed to be normally and independently distributed. Three environments *(B_2016-17*, *B_2017-18*, *and O_2017-18*) were defined as the combination of the sowing season (2016-2017 or 2017-2018) and the trial location (Bretenière or Orsonville). The normality of residuals and homogeneity of variances were checked using Shapiro–Wilk and Bartlett’s test (*P* ≥ 0.05). Broad-sense heritability (*H^2^*) across environments was calculated from ANOVA by $H^{2}=\delta_{\text{g}}^{2}/(\delta_{\text{g}}^{2}+(\delta_{\text{ge}}^{2}/ne)$ , where $\delta_{\text{g}}^{2}$ is the genotypic variance, $\delta_{ge}^{2}$ is the genotype x environment interaction variance, and n_e_ is the number of environments. Analyses were performed in IBM^®^ SPSS Statistics version 26.0.0.0. (https://www.ibm.com/fr-fr/analytics/spss-statistics-software). Principal component analysis (PCA) and Pearson correlation coefficients between traits were calculated using the software Past4.07b (https://www.nhm.uio.no/english/research/infrastructure/past/).

### QTL mapping

Individual genetic maps for *POP2* and *POP3* and the faba bean consensus map described by Carrillo-Perdomo et al. (2020) were used for Quantitative trait loci (QTL) detection. QTLs were identified using MCQTL software v5.2.4 (Jourjon et al., 2005). A composite interval mapping (CIM) and an iterative QTL mapping (iQTL) were carried out. Marker cofactor selection by the forward stepwise method was implemented as well as computation of threshold test values of LOD-scores by 1000 permutations. The ProbaPop component computed QTL genotype probabilities giving marker information at each linkage group for each population (*POP2* and *POP3*). MultiPop detection was performed using the faba bean consensus map developed by Carrillo-Perdomo et al. (2020). Model additive and Interpop connected were used in the MultiPop function to build a pooled QTL model fitting the observations on genotype probabilities for *Pop2* and *Pop3*. MultiPop provided threshold computation, QTL detection methods, and model estimation. The logarithm of the odds (LOD) score, global R^2^, individual R^2^, confidence interval, and allelic effect at each QTL were estimated for each trait and population.

### Comparison of identified QTLs with previous studies in faba bean and other legume species

In order to compare the positions of frost tolerance-related QTLs from this study with those described earlier in faba bean, and in absence of a reference genome sequence for this crop, we took advantage of the bridge markers between the map published by Webb et al. (2016) and our map (Carrillo-Perdomo et al, 2020) as Sallam and Ul-Allah (2019) report QTLs and association peaks anchored to the Webb et al. map. In addition, we used the syntenic relations with *Medicago truncatula* to compare the QTL regions on both maps as both sets of markers have been blasted on the chromosomes of the Mt4.0 model legume genome sequence (Pecrix et al., 2018), https://phytozome-next.jgi.doe.gov/info/Mtruncatula_Mt4_0v1).

Similar to the work done on *M. truncatula*, and in order to search for potential syntenic QTLs from other legume species, these markers were also positioned on the *P. sativum* genome assembly version v.1a (Kreplak et al., 2019) (https://urgi.versailles.inra.fr/Species/Pisum/Pea-Genome-project), on *Cicer arietinum* L. v1 assembly (Varshney et al., 2014) (https://www.ncbi.nlm.nih.gov/assembly/GCF_000331145.1/) and on *Glycine max* L. assembly v2.0 (Schmutz et al., 2010) (http://www.plantgdb.org/GmGDB/) by applying BLASTn searches with marker context sequences and identifying the best match(es) for each sequence in each genome.

## Results

### Faba bean response to freezing temperatures

Two bi-parental RIL populations, namely *POP2* and *POP3*, were evaluated for frost tolerance in three environments (*B_2016-17*, *B_2017-18*, *and O_2017-18*). As expected, the parental lines showed contrasting responses to frost both when considering frost damage (*FD*) and survival rate (*SR*) scores (**Table 1**). The damage caused by up to three frost events (*FD1*, *FD2*, and *FD3*) was scored and the *SR* was noted in each of the three environments and for the two populations (*POP2* and *POP3*) studied. *AUSPC* was calculated to study the evolution of frost damage over time. Environment *B_2016-17* displayed the most extreme conditions (**Figure S1, S3**). A major freezing event took place in January 2017, leading to sixteen successive days of frost with a minimum temperature under shelter close to -10°C and eleven days of ground frost. During the 2017-2018 cropping season, frost events were shorter in duration, both at Bretenière and Orsonville (**Figures S1, S3**). The minimum temperature recorded over the growing period was -11.6°C for the environment *B_2017-18* and -9.5°C in *O_2017-18*, both in February. No negative underground temperatures were recorded for these trials.

**Table 1:** Scores of parental accessions and recombinant inbred lines (RILs) (F_5:6_) from *POP2* (Hiverna x Silian) and *POP3* (Hiverna x Quasar) for the parameters related to faba bean response to freezing temperatures.

Environment^a^	Trait^b^	*Parents*			*POP2*					*POP3*				
		Silian	Hiverna	Quasar	Mean	SD	Min.-Max.	Kurtosis	Skewness	Mean	SD	Min.-Max.	Kurtosis	Skewness
***B_2016-17***	**FD1**	5.00	0.00	1.50	1.82	1.30	0.00-5.00	-1.16	0.21	0.56	0.73	0.00-3.00	2.56	2.64
*** ***	**FD2**	5.00	1.50	4.00	3.99	0.82	1.00-5-00	-0.27	-0.43	2.93	0.88	0.00-5.00	0.39	0.39
*** ***	**FD3**	5.00	2.00	4.50	4.30	0.79	1.00-5.00	0.36	-0.91	3.21	0.98	1.00-5.00	-0.50	-0.39
*** ***	**AUSPC**	422.50	140.00	351.25	323.54	58.50	84.00-422.00	0.71	-0.67	237.45	72.92	53.00-401.00	-0.32	0.81
*** ***	**SR**	0.00	59.50	7.00	14.95	20.08	0-00-94.00	2.03	1.52	44.71	26.09	0.00-100.00	-0.87	-0.70
														
***B_2017-18***	**FD1**	3.50	0.00	0.00	0.21	0.25	0.00-0.50	-1.90	5.14	0.09	0.19	0.00-0.50	0.80	1.69
*** ***	**FD2**	5.00	1.00	1.50	1.91	0.71	0.00-4.00	-0.67	10.63	1.43	0.53	0.00-2.00	-1.27	-0.01
*** ***	**AUSPC**	288.00	24.00	36.00	54.25	38.50	0.00-552.00	94.38	6.51	34.21	17.06	0.00-72.00	-0.78	0.09
*** ***	**SR**	0.00	87.00	100.00	78.46	19.65	5.00-100.00	0.97	-1.27	92.57	7.75	67.00-100.00	0.28	-0.95
														
***O_2017-18***	**FD1**	4.00	2.50	2.00	2.84	0.79	1.00-5.00	-0.12	-0.09	2.27	0.71	1.00-4.00	-0.21	0.12
*** ***	**SR**	0.00	100.00	94.00	91.34	11.48	42.00-100.00	3.76	-3.01	97.06	6.87	43.00-100.00	23.67	-4.12

a B_2016-17, Bretenière in 2016-2017; B_2017-18, Bretenière in 2017-2018; O_2017-18, Orsonville in 2017-2018.

b FD1, first rating of symptoms of frost damage (0-5); FD2, second rating of symptoms of frost damage (0-5); FD3, third rating of symptoms of frost damage (0-5); AUSPC, area under the symptoms progress curve calculated from the ratings of FD1, FD2 and FD3 and; SR, survival rate after the frost events.

In general, the susceptible parent Silian showed higher *FDs* and *AUSPC* scores and a lower *SR* than Hiverna and Quasar. Thus, Silian seems to present a higher sensitivity to frost damage as compared to Hiverna and Quasar. No significant differences (P>0.05) were found between Hiverna and Quasar in the environment *O_2017-18.* Hiverna presented a higher tolerance to frost and ability to survive than Quasar during the 2016-2017 crop season in Bretenière (*FD2*, *FD3*, and *PS* in *B_2016-17* and *FD1* in *B_2017-18*) (**Table 1**). Nonetheless, the extreme frost event of January 2017 reduced the survival rate of the tolerant parents Hiverna and Quasar in the environment *B_2016-17*.

The frequency distribution of *FD1*, *FD2, FD3*, and *SR* in the RIL populations *POP2* and *POP3* showed a continuous non-normal distribution in the three environments (**Table 1**; **Figure S3**). Several transgressive RILs had higher tolerance to frost and survival rates than the tolerant parents Hiverna and Quasar. One line of *POP2* (VFP-2-0734) and three lines of *POP3* (VFP-3-0307, -1211, -1281) presented higher or similar levels of frost tolerance and survival regarding the tolerant parent Hiverna. These four lines presented stable levels of tolerance to frost and survival throughout the three environments. No lines more sensitive to frost than the susceptible parent Silian were identified.

**Figure 1:**
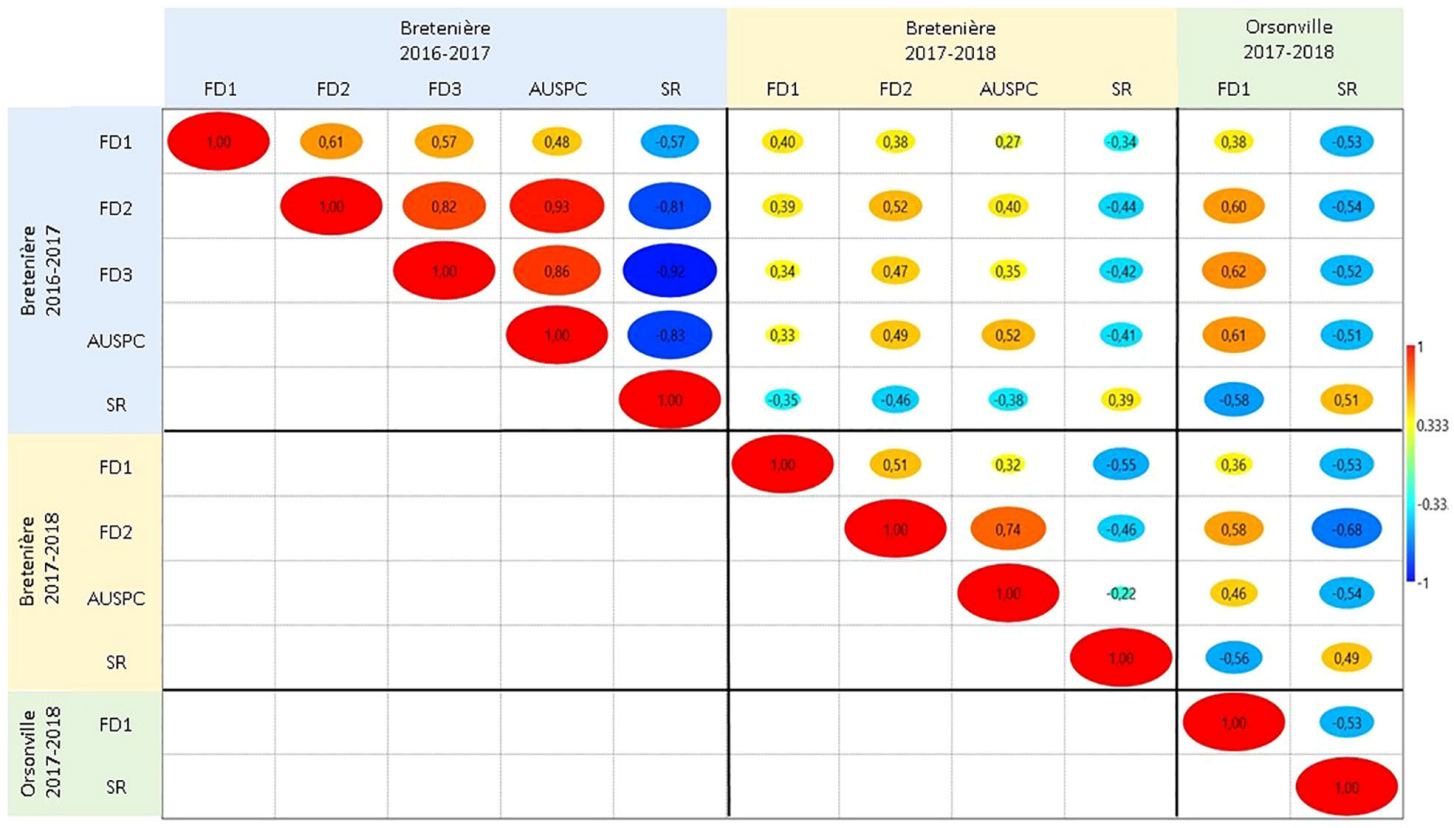
Pearson correlation between the traits scored in the two faba bean (Vicia faba) recombinant inbred line (RIL) populations *POP2* (Hiverna x Silian) and *POP3* (Hiverna x Quasar) in the three environments studied: Bretenière during the 2016-2017 cropping season, Bretenière during the 2017-2018 cropping season and Orsonville during the 2017-2018 cropping season. Cold colors indicate negative correlations while warm colors indicate positive correlations between traits. FD1 stands for plant damage caused by the first frost event. FD2 refers to plant damage after the second frost event. FD3 corresponds to plant damage after the third frost event. AUSPC is the area under the symptoms progress curve after the frost period. SR stands for survival rate after the frost period.

**Figure 2:**
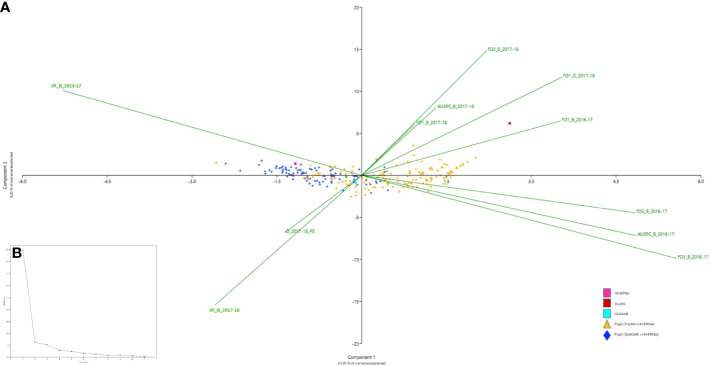
Principal component analysis (PCA) of the individuals of the two faba bean recombinant inbred line (RIL) populations *POP2* (Hiverna x Silian) and *POP3* (Hiverna x Quasar) and the parental lines Hiverna, Silian and Quasar and the scored variables: FD1, plant damage caused by the first frost event, FD2, plant damage after the second frost event FD3, plant damage after the third frost event, AUSPC, area under the symptoms progress curve after the frost period and SR, survival rate after the frost period; in the different environments studied (B_2016-17, Bretenière during the 2016-2017 cropping season, B_2017-18 Bretenière during the 2017-2018 cropping season and O_2017-18, Orsonville during the 2017-2018 cropping season). **(A)** Biplot of the first (x-axis) and second (y-axis) principal components (PCs) that shows the PCA scores of the explanatory variables as vectors (in green) and individuals (*POP2* in orange, *POP3* in dark blue, Hiverna in pink, Silian in burgundy and Quasar in light blue). Individuals on the same side as a given variable should be interpreted as having a high contribution on it. The magnitude of the vectors (lines) shows the strength of their contribution to each PC. Vectors pointing in similar directions indicate positively correlated variables, vectors pointing in opposite directions indicate negatively correlated variables, and vectors at proximately right angles indicate low or no correlation. **(B)** Scree plot of eigenvalues after PCA.

Broad-sense heritability ranged from 0.51 (*FD1*) to 0.85 (*FD2*) in *POP2* and from 0.24 (*FD3*) to 0.78 (*FD2)* in *POP3* (**Table 2**). Pearson correlations between the traits, by environment and by population, are shown in **Figures 1** and **S3**. In general, we observed positive correlations among different frost damage scores and negative correlations between frost damage scores and plant survival rate. This trend was confirmed by the Principal Component (PC) Analysis (**Figure 2**). PC1 captured 67.1% of the variation, allowing for differentiation between frost tolerant and susceptible accessions (**Figure 2**); while PC2 captured 9.2% of the variation, allowing for differentiation of the plant responses to frost in the different environments studied during the 2016-2017 and 2017-2018 cropping seasons.

**Table 2:** Broad-sense heritability (*H^2^*) of the traits scored in the recombinant inbred lines (RILs) (F_5:6_) from *POP2* (Hiverna x Silian) and *POP3* (Hiverna x Quasar).

Trait^a^	*POP2*	*POP3*
FD1	0.51	0.31
FD2	0.85	0.78
FD3	0.65	0.24
AUSPC	0.77	0.44
SR	0.77	0.69

a FD1, first rating of symptoms of frost damage; FD2, second rating of symptoms of frost damage; FD3, third rating of symptoms of frost damage; AUSPC area under the symptoms progress curve calculated from the ratings of FD1, FD2 and FD3 and; SR survival rate after the frost events.

### Faba bean QTLs for frost damage and survival rate

**Figure 3:**
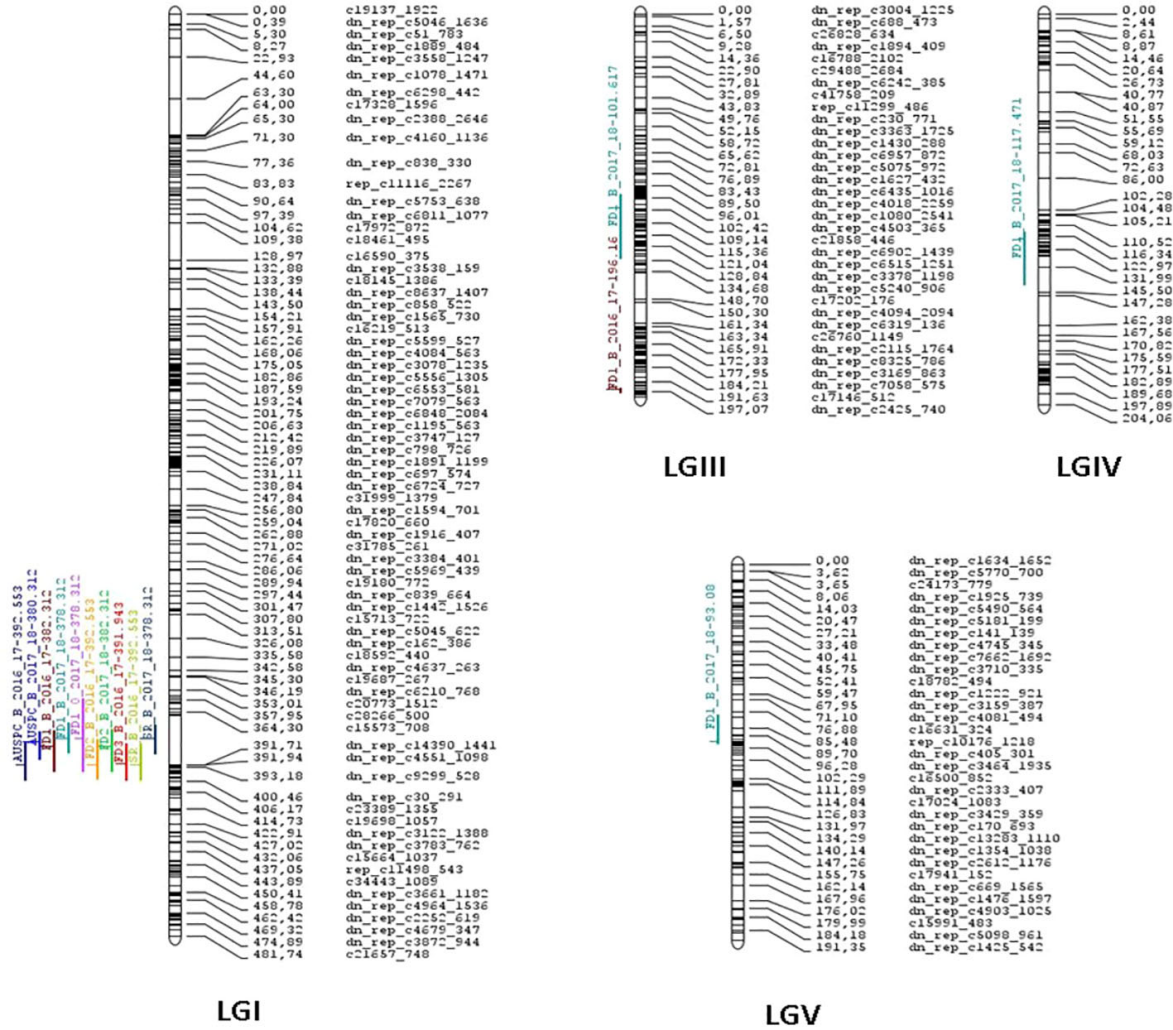
Faba bean (Vicia faba) consensus genetic linkage map constructed from two populations formed by 145 F5:6 recombinant inbred lines (RILs) derived from the cross between Hiverna x Silian and 96 RILs F5:6 derived from the cross Hiverna x Quasar. Colored lines on the left of chromosome bars indicate the locations of quantitative trait loci (QTLs). The lengths of the lines are proportional to the lengths of the respective confidence intervals.

QTL analysis using CIM and iQTL mapping revealed consistent genomic regions associated with frost damage, i.e. *FD1, FD2, FD3*, and *AUSPC*, and plant survival (*SR*) in the three environments *(B_2016-17, B_2017-18*, and *O_2017-18).* (**Table 3**; **Figures 3**, **S4**). Five QTLs were identified along LG I, III, IV, and V. On one hand, the QTLs involved in plant tolerance to the damage caused by frost events explained individually from 8.37 to 27.60% of the total phenotypic variance, depending on the trait scored. They explained together from 12.96 to 58.26% of the phenotypic variance. On the other hand, the QTLs for plant survival rate explained from 17.22 to 24.37% of the phenotypic variance. Linkage group I contained a robust and consistent meta-QTL detected in *POP2* in all environments for frost damage scores and survival rate. It accounts for 12.96 to 27.60% of the phenotypic variance of frost damage scores and for 17.22 to 24.37% of survival rate variance. The meta-QTL is located between the gene-based SNP markers “dn_rep_c2728_1677” and “dn_rep_c30_291” and individual QTLs peaked around 378.31 to 392.55 cM with LOD values from 7.05 to 16.41, depending on the trait. QTLs identified in LGIII, LGIV and LGV were only detected in the two trials located in Bretenière. In LGIII, two QTLs associated to reduced damage after the first frost event were identified in both *POP2 and POP3*: the QTL *FD_III.1_FD1_B_17-18* identified during the 2017-2018 cropping season and the QTL *FD_III.2_FD1_B_16-17* during 2016-2017. *FD_III.1_FD1_B_17-18* accounts for 8.37% of phenotypic variance and is located between “dn_rep_c879_1279” and “dn_rep_c3378_1198” markers with a peak at 101.62 cM (LOD score of 4.33). *FD_III.2_FD1_B_16-17* accounts for 22.59% of phenotypic variance and is located between “dn_rep_c125_455” and “dn_rep_c3943_1691” markers with a peak at 196.16 cM (LOD score of 12.89). In LGIV, the QTL *FD_IV.1_FD1_B_17-18* is associated with reduced damage after the first frost event occurring during the 2017-2018 cropping season. It was only detected in *POP3* and accounts for 9.22% of the phenotypic variance. It is located between “dn_rep_c3237_978” and “dn_rep_c6181_713” markers with a peak at 117.47 cM (LOD score of 4.79). Finally, the QTL *FD_V.1 FD1_B_17-18* was identified on LGV in *POP2* and *POP3.* It accounts for 12.99% of the phenotypic variance associated with reduced frost damage during the first frost event. It is located between “dn_rep_c3712_1645” and “dn_rep_c958_552” markers with a peak at 93.08 cM and a LOD score of 6.89.

**Table 3:** Quantitative trait loci (QTL) for frost tolerance.

Trait^a^		LG^b^	QTL name	Peak position^c^	LOD^d^	Inferior marker^e^	Peak marker^f^	Superior marker^g^	*POP2* Hiverna x Silian	*POP3 *Hiverna x Quasar	Add^he^ Silian	Add^if^ Hiverna	Add^jg^ Quasar	R^2kh^
***FD1_B_2016-17***		I	*FD_I.1_FD1_B_16-17*	382.31	13.41	c16955_467	c16955_467	dn_rep_c9956_437	X		0.36	-0.24	-0.12	0.23
***FD1_B_2016-17***		III	*FD_III.2_FD1_B_16-17*	196.16	12.90	dn_rep_c125_455	dn_rep_c2278_599	dn_rep_c3943_1691	X	X	0.31	-0.19	-0.12	0.23
	*Total*													*0.46*
***FD1_B_2017-18***		I	*FD_I.1_FD1_B_17-18*	378.31	12.85	c16955_467	c16955_467	dn_rep_c14390_1441	X		0.09	-0.05	-0.04	0.23
***FD1_B_2017-18***		III	*FD_III.1_FD1_B_17-18*	101.62	4.33	dn_rep_c879_1279	rep_c11396_777	dn_rep_c3378_1198	X	X	0.04	-0.02	-0.02	0.08
***FD1_B_2017-18***		IV	*FD_IV.1_FD1_B_17-18*	117.47	4.79	dn_rep_c3237_978	c17139_182	dn_rep_c6181_713		X	0.02	0.03	-0.05	0.09
***FD1_B_2017-18***		V	*FD_V.1_FD1_B_17-18*	93.08	6.89	dn_rep_c3712_1645	rep_c10474_1278	dn_rep_c958_552	X	X	0.05	-0.02	-0.03	0.13
	*Total*													*0.53*
***FD1_O_2017-18***		I	*FD_I.1_FD1_O_17-18*	378.31	7.05	dn_rep_c2728_1677	c16955_467	dn_rep_c3119_1300	X		0.23	-0.17	-0.05	0.13
	*Total*													*0.13*
***FD2_B_2016-17***		I	*FD_I.1_FD2_B_16-17*	392.55	9.64	c16955_467	dn_rep_c8507_205	dn_rep_c30_291	X		0.20	-0.16	-0.04	0.17
***FD2_B_2017-18***		I	*FD_I.1_FD2_B_17-18*	382.31	12.04	c16955_467	c16955_467	dn_rep_c3119_1300	X		0.28	-0.14	-0.14	0.21
	*Total*													*0.38*
***FD3_B_2016-17***		I	*FD_I.1_FD3_B_16-17*	391.94	9.83	dn_rep_c14390_1441	dn_rep_c4551_1098	dn_rep_c30_291	X		0.17	-0.17	-0.01	0.18
	*Total*													*0.18*
***AUSPC_B_2016-17***		I	*FD_I.1_AUSPC_B_16-17*	392.55	8.45	dn_rep_c14390_1441	dn_rep_c8507_205	dn_rep_c30_291	X		12.97	-11.29	-1.67	0.15
	*Total*													*0.15*
***AUSPC_B_2017-18***		I	*FD_I.1_AUSPC_B_17-18*	380.31	16.41	c16955_467	c16955_467	dn_rep_c14390_1441	X		9.17	-4.47	-4.70	0.28
	*Total*													*0.28*
***SR_B_2016-17***		I	*SR_I.1_PS_B_16-17*	392.55	9.60	dn_rep_c14390_1441	dn_rep_c8507_205	dn_rep_c30_291	X		-5.70	3.84	1.86	0.17
	*Total*													*0.17*
***SR_B_2017-18***		I	*SR_I.1_PS_B_17-18*	378.31	14.19	c16955_467	c16955_467	c16955_467	X		-7.91	3.62	4.29	0.24
	*Total*													*0.24*

a FD1_B_2016-17 first rating of symptoms of frost damage (FD1) in the trial of the Époisses experimental farm in Bretenière during 2016-2017 sowing season (B_2016-17), FD1_B_2017-18 first rating of symptoms of frost damage (FD1) in the trial at Bretenière during 2017-2018 sowing season (B_2017-18), FD1_O_2017-18 first rating of symptoms of frost damage (FD1) in the trial at Orsonville during 2017-2018 sowing season (O_2017-18); FD2_B_2016-17 second rating of symptoms of frost damage (FD2) in the trial at Bretenière during 2016-2017 sowing season (B_2016-17), FD2_B_2017-18 second rating of symptoms of frost damage (FD2) in the trial at Bretenière during 2017-2018 sowing season (B_2017-18), FD3_B_2016-17 third rating of symptoms of frost damage (FD3) in the trial at Bretenière during 2016-2017 sowing season (B_2016-17), AUSPC_B_2016-17 area under the symptoms progress curve calculated from the ratings of FD1, FD2 and FD3 in the trial of Bretenière during 2016-2017 sowing season (B_2016-17), AUSPC_B_2017-18 area under the symptoms progress curve calculated from the ratings of FD1 and FD2 in the trial at Bretenière during 2017-2018 sowing season (B_2017-18), SR_B_2016-17 survival rate (SR) in the trial at Bretenière during 2016-2017 sowing season (B_2016-17) and SR_B_2017-18, SR in the trial of Bretenière during 2017-2018 sowing season (B_2017-18),

b LG, linkage group.

c Peak position of the QTL (cM).

d LOD the peak LOD score.

e Inferior marker, molecular marker that coincides with the lowest position of the confidence interval of the QTL.

f Peak marker, molecular marker that coincides with the position in the confidence interval of the QTL at which the LOD score value is maximum.

g Superior marker, molecular marker that coincides with the uppest position of the confidence interval of the QTL.

h Add Silian the additive effect of the multicross Silian-Hiverna-QUASAR regarding the parent Silian.

i Add Hiverna the additive effect of the multicross Silian-HIVERNA-QUASAR regarding the parent HIVERNA.

j Add Quasar the additive effect of the multicross Silian-HIVERNA-QUASAR regarding the parent QUASAR.

k R^2^ proportion of the phenotypic variance explained by the respective QTL.

The desirable alleles for coping with frost tolerance are mainly derived from the parents Hiverna and Quasar (**Table 3**).

### Co-localization of frost tolerance QTLs in different faba bean genetic pools

Previous studies have reported the identification of faba bean QTLs for frost tolerance (Arbaoui et al., 2008a; Sallam and Martsch, 2015; Sallam et al., 2016a; Sallam et al., 2016b). Among them, Sallam et al. (Sallam and Martsch, 2015; Sallam et al., 2015; Sallam et al., 2016b) used the SNP-based consensus map developed by Webb et al. (Webb et al., 2016) for QTL and association mapping, making possible the comparison with our results. Sallam and Martsch (2015) and Sallam et al. (2016b) also identified a QTL region associated with frost tolerance and fatty acid composition at the bottom of LGI (QTL region flanked by the markers VF_Mt5g026780 and VF_Mt2g027240). This QTL seems to co-localize with the major frost tolerance QTL (*FD_I.1* and *SR_I.1*) identified in the present study. Moreover, the environment-specific QTLs *FD_III.1, FD_III.*2, *FD_IV.1* and *FD_V.1* (identified in LGIII, IV, and V, respectively) seem to be located in between QTLs involved in frost tolerance and fatty acid composition previously identified on LGIII around 78 cM (Sallam and Martsch, 2015) and 136 cM (Sallam and Martsch, 2015; Sallam and Martsch, 2016; Sallam et al., 2016b), with a frost tolerance QTL of LGIV with a peak around 129 cM (Sallam and Martsch, 2015) and in between proline and fatty acid composition QTLs at 57 (Sallam et al., 2016b), 67 (Sallam and Martsch, 2016; Sallam et al., 2016b) and 73 (Sallam and Martsch, 2016; Sallam et al., 2016b) cM on LGV [see Sallam and Ul-Allah (2019)].

### Synteny between genomic regions harboring QTLs for frost tolerance in faba bean and in close legume species

Taking advantage of the syntenic relationship between legume species, the positions of the QTLs identified in this study were compared with those QTLs of frost damage previously reported in other legume species (Kahraman et al., 2004; Lejeune-Hénaut et al., 2008; Dumont et al., 2009; Zhang et al., 2011; Zhang et al., 2012; Avia et al., 2013; Tayeh et al., 2013a; Kahraman et al., 2015; Ates et al., 2018; Deokar et al., 2019; Mugabe et al., 2019; Beji et al., 2020). The faba bean major QTL on LGI (*FD_I.1* and *SR_I.1*) is syntenic to the major freezing tolerance QTLs of chromosome 6 of *Medicago truncatula* (Tayeh et al., 2013a) and of LGVI of *Pisum sativum* (Lejeune-Hénaut et al., 2008) (**Table S1**). Interestingly, although the QTLs *FD_IV.1* (LGIV) and *FD_V.1* (LGV) are environment-specific, they seem to correspond to frost tolerance QTLs previously reported in other legume species. So, the QTL *FD_IV.1* appeared to be syntenic to *Sat_249* (Zhang et al., 2011) of LGJ of soybean (*Glycine max* L.) while *FD_V.1* is potentially syntenic to QTL *V.2* reported on LGV of pea (Dumont et al., 2009; Klein et al., 2014; Beji et al., 2020) and to *Sat_126* (Zhang et al., 2012) (LGK) of soybean (**Table S1**).

## Discussion

### Progress towards improving frost tolerance in faba bean

Autumn-sown faba beans are known to be more productive than spring types (Sallam et al., 2016b; Landry and Hu, 2019). However, yield stability across environments continues to be a limitation that needs to be tackled. The development of frost-tolerant varieties with stable yields is especially critical to ensure productivity, so that, even if the temperatures seriously decrease, the physiological impact on the plants does not translate into a significant reduction in their yield. This is especially important when dealing with climate change since the incidence of extreme climatic events is becoming more frequent. Our results, together with other efforts to decipher frost tolerance genetics in winter faba bean (Arbaoui et al., 2008a; Sallam and Martsch, 2015; Sallam et al., 2015; Sallam et al., 2016), open avenues for breeding improved varieties. As expected, the level of tolerance and survival to frost along the RILs families of both populations was affected by the environmental conditions. This has also been previously reported in other faba been genetic pools (Arbaoui et al., 2008b). Nonetheless, four RILs [one line of *POP2* (VFP-2-0734) and three of *POP3* (VFP-3-0307, -1211, -1281)] presented high and stable levels of tolerance to frost. These lines are available for faba bean breeding programs.) Moreover, the identification of a major QTL controlling frost tolerance on the LGI (*FD_I.1* and *SR_I.1*) holds promise for MAS development (**Table 3**). Favorable alleles for frost tolerance mainly came from Hiverna and Quasar (**Table 3**). Interestingly, the recombinant inbred lines VFP-2-0734, VFP-3-0307, VFP-3-1211 and VFP-3-1281 cumulated several frost damage and/or plant survival QTLs, which makes them good genitors for faba bean breeding programs. Pyramiding favorable alleles from Quasar and Hiverna should increase the chances of coping with sub-zero temperatures by accumulating genes involved in different mechanisms for acclimatizing and de-acclimating to sub-zero temperatures, as suggested by progenies showing transgressive performances.

### Some genomic regions of frost tolerance are common among different faba bean genetic backgrounds

The major QTL on LGI harboring *FD_I.1* and *SR_I.1*, co-localizes with the genomic region involved in frost tolerance and fatty acid composition previously described in Sallam and Martsch (2015) and in Sallam et al. (2016a). Sallam et al. (2016b) confirmed several frost tolerance QTLs (Sallam et al., 2015) by association mapping using 156 gene-based SNP markers (Webb et al., 2016), confirming their stability in different environments and genetic backgrounds. The authors used the synteny between the faba bean genetic map and the genome sequence of the model legume *Medicago truncatula* L. (Webb et al., 2016) to investigate the genomic regions involved in the faba bean response to frost. Thus, *FD_I.1* and *SR_I.1* are robust QTLs of utility for MAS. We also identified (only in Bretenière) QTLs on LGIII (*FD_III.1* and *FTIII.2*), LGIV (*FD_IV.1*), and LGV (*FD_V.1*) (**Table 3**). These QTLs co-localize with faba bean frost tolerance QTLs previously reported on LGIII (Sallam and Martsch, 2015; Sallam and Martsch, 2016; Sallam et al., 2016b), LGIV (Sallam and Martsch, 2015), and LGV (Sallam et al., 2016b) from different populations and different trials.

### Some frost tolerance genomic regions are conserved among legume species

The solid blocks of macrosynteny between faba bean and closely-related legume species^22,23^ (i.e. pea, barrel medic, or chickpea (Webb et al., 2016; Carrillo-Perdomo et al., 2020), but also with divergent legume species (Carrillo-Perdomo et al., 2020) [i.e. common bean (*Phaseoulus vulgaris* L.), soybean or birdsfoot trefoil (*Lotus japonicus* L.)] facilitates comparative genomic studies that allow the exploration of gene and QTL conservation among legumes. Genomic regions harboring syntenic QTLs between faba bean and other legume species have been reported for flowering and pod setting (Cruz-Izquierdo et al., 2012), where QTL were conserved among faba bean, *M. truncatula*, pea, lupine (*Lupinus albus* L.), chickpea (*C. arietinum*) and birdsfoot trefoil; or for resistance to *Ascochyta fabae* Speg. and *Orobanche crenata* Forssk., where QTLs (Gutierrez and Torres, 2021) were syntenic between faba bean and barrel medic. The comparison of the positions of QTLs reported in this study and those of previously reported QTLs for frost tolerance in other legume species (Kahraman et al., 2004; Lejeune-Hénaut et al., 2008; Dumont et al., 2009; Zhang et al., 2011; Zhang et al., 2012; Avia et al., 2013; Tayeh et al., 2013b; Kahraman et al., 2015; Ates et al., 2018; Deokar et al., 2019; Mugabe et al., 2019; Beji et al., 2020), showed some conserved genomic regions. The faba bean major QTL *FD_I.1* (LGI) is syntenic with the major freezing tolerance QTLs reported in barrel medic (Tayeh et al., 2013b) on chromosome 6 and in pea on LGVI (Lejeune-Hénaut et al., 2008). Moreover, *FD_IV.1 QTL* (LGIV) seems to be syntenic to *Sat_249* (Zhang et al., 2011) described in LGJ of soybean and *FD_V.1* (LGV) is syntenic to the frost tolerance QTLs *V.2* reported in pea on LGV (Dumont et al., 2009; Klein et al., 2014; Beji et al., 2020) and with *Sat_126* present on LGK of soybean (Zhang et al., 2012). These results further support the functional conservation of key genetic determinants for agronomic traits between legumes. Exploiting this conservation could make the identification of potential candidate genes more agile.

The region of chromosome 6 of barrel medic (Tayeh et al., 2013b) named Mt-FTQTL6 syntenic to the faba bean QTL *FD_I.1* contains twelve C-repeat binding factor (CBF)/dehydration-responsive element binding factor 1 (DREB1) genes organized in a tandem array. This QTL region is also collinear with a frost damage QTL of LGVI of pea (Tayeh et al., 2013a) and was in addition revealed by a Genome-Wide approach, which pinpointed CBF genes at the vicinity of frost-damage association peaks (Dumont et al., 2009). The CBF/DREB1 transcription factors have been shown to play a major role in plant cold acclimation in the model plant *Arabidopsis thaliana* (Alonso-Blanco et al., 2005; Thomashow, 2010) and to co-localize with freezing tolerance QTLs in other species (Vágújfalvi et al., 2003; Knox et al., 2008; Sandve et al., 2011). In durum wheat, (Sieber et al., 2016) showed that frost tolerance is mainly controlled by a *CBF* copy number variation at the *Fr* locus, a main frost resistance QTL of the *Poaceae*. In *Triticum monococcum*, studies of lines carrying recombination events within the homologous CBF cluster evidenced a non-functional CBF copy in a more sensitive line and three copies allowing to cold acclimate at milder temperatures in a more tolerant line (Knox et al., 2008).In addition, mutational approaches have evidenced the role of CBF regulons in cold acclimation in *Arabidopsis thaliana* (Jia et al., 2016; Zhao et al., 2016). The ongoing genomic and pan genomic approaches in faba bean (Khazaei et al., 2015) will allow establishing the presence of a CBF regulon in the QTL *FD_I.1* region and will enable the study of the organization and polymorphism of the locus in faba bean accessions with contrasted phenotypes for frost tolerance. The same synteny-based approach could be applied to explore the gene content of the other QTLs evidenced in this study.

In conclusion, four novel RILs showing high levels of tolerance and ability to recover from freezing temperatures by accumulating frost tolerance QTLs are now available for breeding programs. This work has demonstrated the suitability of high-density gene-based genetic maps (Carrillo-Perdomo et al., 2020) to identify QTLs with relatively small confidence intervals and exploit syntenic relationships between legumes in order to validate genomic regions involved in frost tolerance. It also highlighted the conservation of the genetic control of frost tolerance among different faba bean genetic pools and legume species, showing the relevance of translational approaches. The development of pan genomic sequencing in legumes will open new perspectives to tackle the understanding of traits as complex as frost tolerance.

## Data availability statement

The datasets presented in this study can be found in online repositories. The names of the repository/repositories and accession number(s) can be found in the article/**Supplementary Material**.

## Author contributions

EC-P analyzed the data, performed the QTL and synteny analysis and wrote the manuscript; JM-R, MF, BR, and CD performed the phenotyping; PM developed the recombinant populations and conceived the experiment; JB, IL-H, and NT edited and critically reviewed the manuscript; GA conceived and supervised the experiments, analyzed the data, performed the synteny analysis, edited and reviewed the manuscript. All authors read and approved the manuscript.

## Funding

This research was supported by the PeaMUST project, which was funded by the French government through the Investment for the Future program (project ANR-11-BTBR-0002) and Ministère de l’Enseignement supérieur, de la Recherche et de l’Innovation.

## Acknowledgments

Authors thank the field and laboratory staff at INRAE and Agri Obtentions for technical assistance and for their participation in the production of the recombinant inbred line populations and phenotyping.

## Conflict of interest

The authors declare that the research was conducted in the absence of any commercial or financial relationships that could be construed as a potential conflict of interest.

## Publisher’s note

All claims expressed in this article are solely those of the authors and do not necessarily represent those of their affiliated organizations, or those of the publisher, the editors and the reviewers. Any product that may be evaluated in this article, or claim that may be made by its manufacturer, is not guaranteed or endorsed by the publisher.
