# Supplementary material for: A QTL approach in faba bean highlights the conservation of genetic control of frost tolerance among legume species
Source: Front Plant Sci. 2022 Oct 19;13:970865. doi: 10.3389/fpls.2022.970865 (PMC9627038; doi:10.3389/fpls.2022.970865)
Supplement: Supplementary file 1 [file DataSheet_1.pdf]

Table S1. Description of the climatic parameters recorded in the three environments studied.

Environment	Location	Sowing seas on	T_{max}[a] (°C)	T_{min}[b] (°C)	AvT[c] (°C)	H_{min}[d] (%)	H_{max}[e] (%)	Rainfall (mm)	S_{max}_Win d[f] (km/h)	AvS Wind[g] (km/h)	Global horizontal irradiance (J/m²)	Insolation duration (h)
B_2016-17	Breteniѐre	2016-2017	21.60	-9.60	6.61	75.18	95.06	1.39	7.01	2.04	558.91	3.32
B_2017-18	Breteniѐre	2017-2018	12.00	-11.60	2.74	74.07	96.73	2.75	8.42	2.45	618.85	2.70
O_2017-18	Orsonville	2017-2018	18.7	-9.50	6.72	94.39	71.02	2.32	11.11	3.15	431.39	2.18

[a] *Tmax* Maximum temperature.
[b] *Tmin* Minimum temperature.
[c] *AvT* Average temperature.
[d] *Hmin* Minimum relative humidity.
[e] *Hmax* Maximum relative humidity.
[f] *Smax Wind* Maximum speed of wind.
[g] *AvSWind* Average speed of wind.
