# Supplementary material for: A QTL approach in faba bean highlights the conservation of genetic control of frost tolerance among legume species
Source: Front Plant Sci. 2022 Oct 19;13:970865. doi: 10.3389/fpls.2022.970865 (PMC9627038; doi:10.3389/fpls.2022.970865)
Supplement: Supplementary file 2 [file DataSheet_2.pdf]

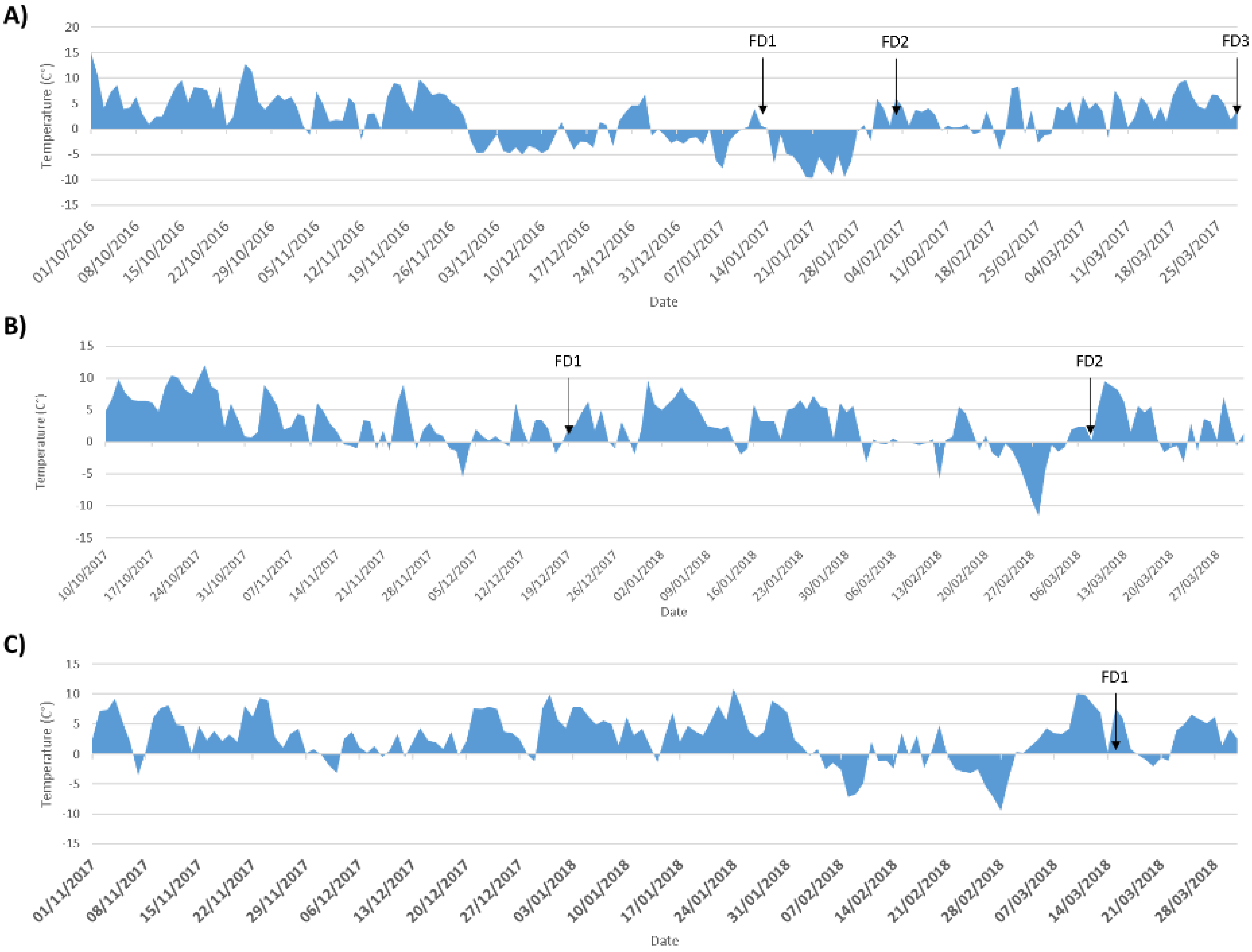

A)
FD1
FD2
FD3
Temperature (C°)
Date
B)
FD1
FD2
Temperature (C°)
Date
C)
FD1
Temperature (C°)
Date

Figure S1. Evolution of daily temperature in the environments where the faba bean (*Vicia faba L.*) recombinant inbred line (RIL) populations *Pop2* (Hiverna x Silian) and *Pop3* (Hiverna x Quasar) were evaluated for frost tolerance. A) Bretenière during the 2016-2017 cropping season, B) Bretenière during the 2017-2018 cropping season and C) Orsonville during the 2017-2018 cropping season. Only the periods when frost is likely to occur are considered for the graphs. *FD1* stands for plant damage caused by the first frost event. *FD2* refers to plant damage after the second frost event. *FD3* corresponds to plant damage after the third frost event.

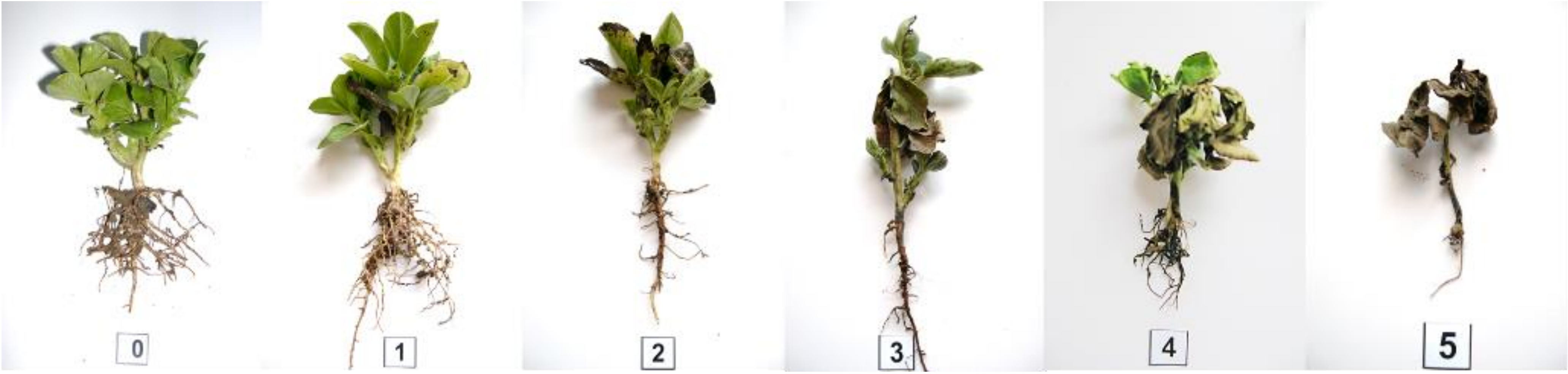

Figure S2. Visual scale from zero to five used to evaluate the symptoms of frost damage. Zero corresponds to no visual damage and five corresponds to dead plant.

A)

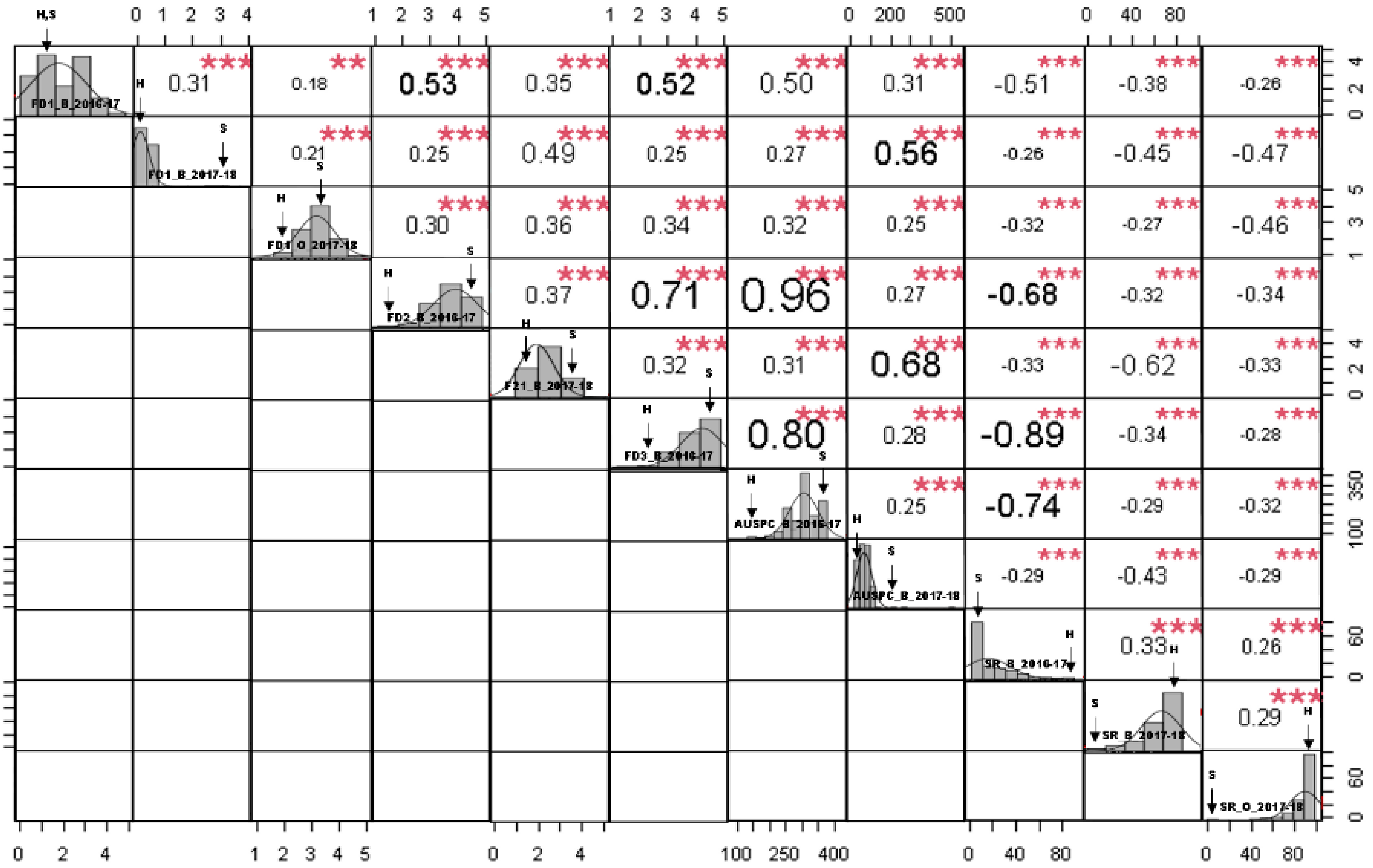

FD1_B_2016-17
FD1_B_2017-18
FD1_O_2017-18
FD2_B_2016-17
F21_B_2017-18
FD3_B_2016-17
AUSPC_B_2016-17
AUSPC_B_2017-18
SR_B_2016-17
SR_B_2017-18
SR_O_2017-18

B)

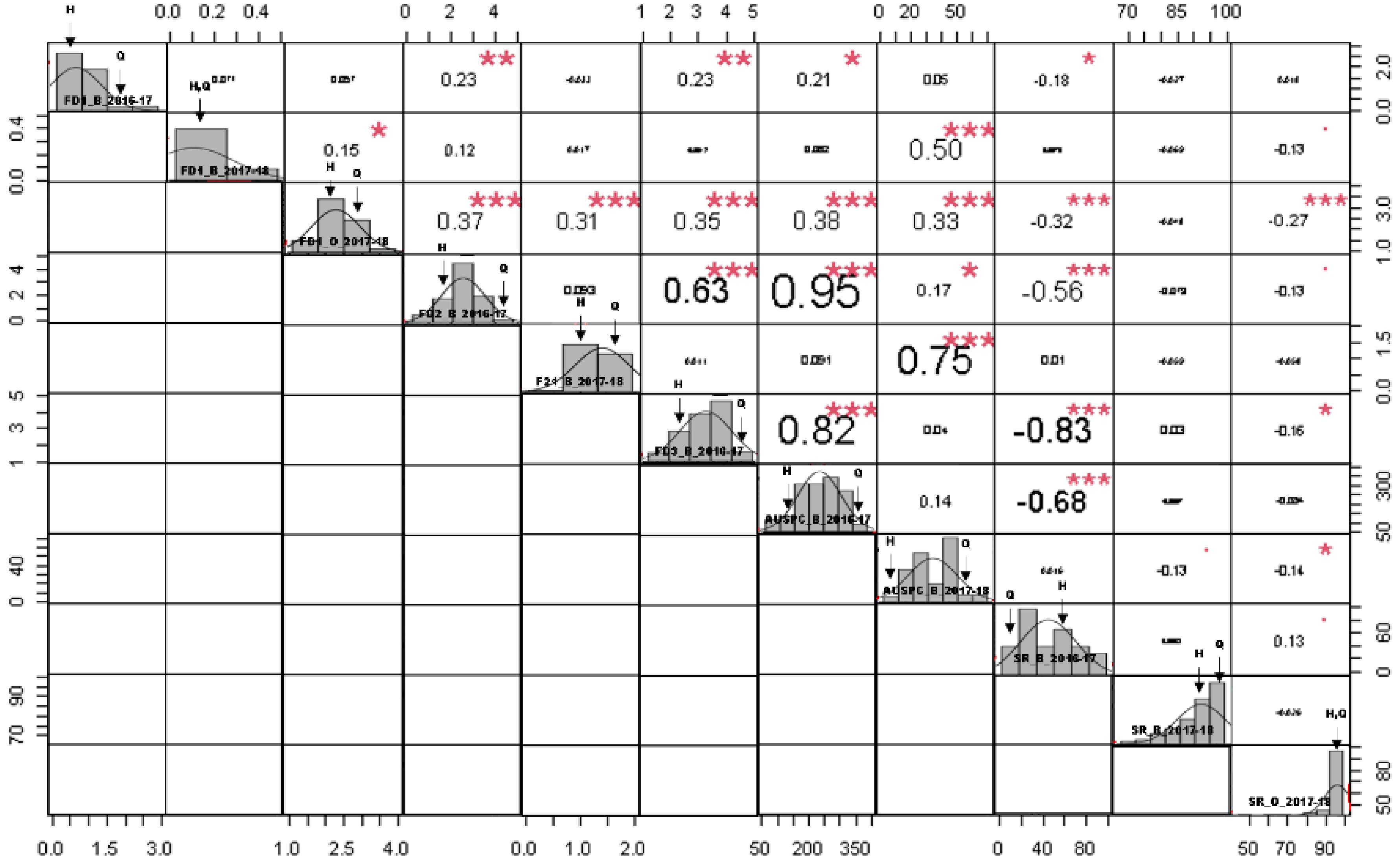

Figure S3. Pearson correlations (upper pannel) and frequency distribution (diagonal pannel) of frost damage (*FD1*, plant damage caused by the first frost event; *FD2*, plant damage after the second frost event; *FD3*, plant damage after the third frost event and *AUSPC*, area under the symptoms progress curve) and

survival rate (*SR*) of the parents Hiverna (*H*), Silian (*S*) and Quasar (*Q*) and the recombinant inbred lines (RILs) ($F_{5:6}$) from A) *Pop2* (Hiverna x Silian) and B) *Pop3* (Hiverna x Quasar). *Asterisks* indicate significative differences.

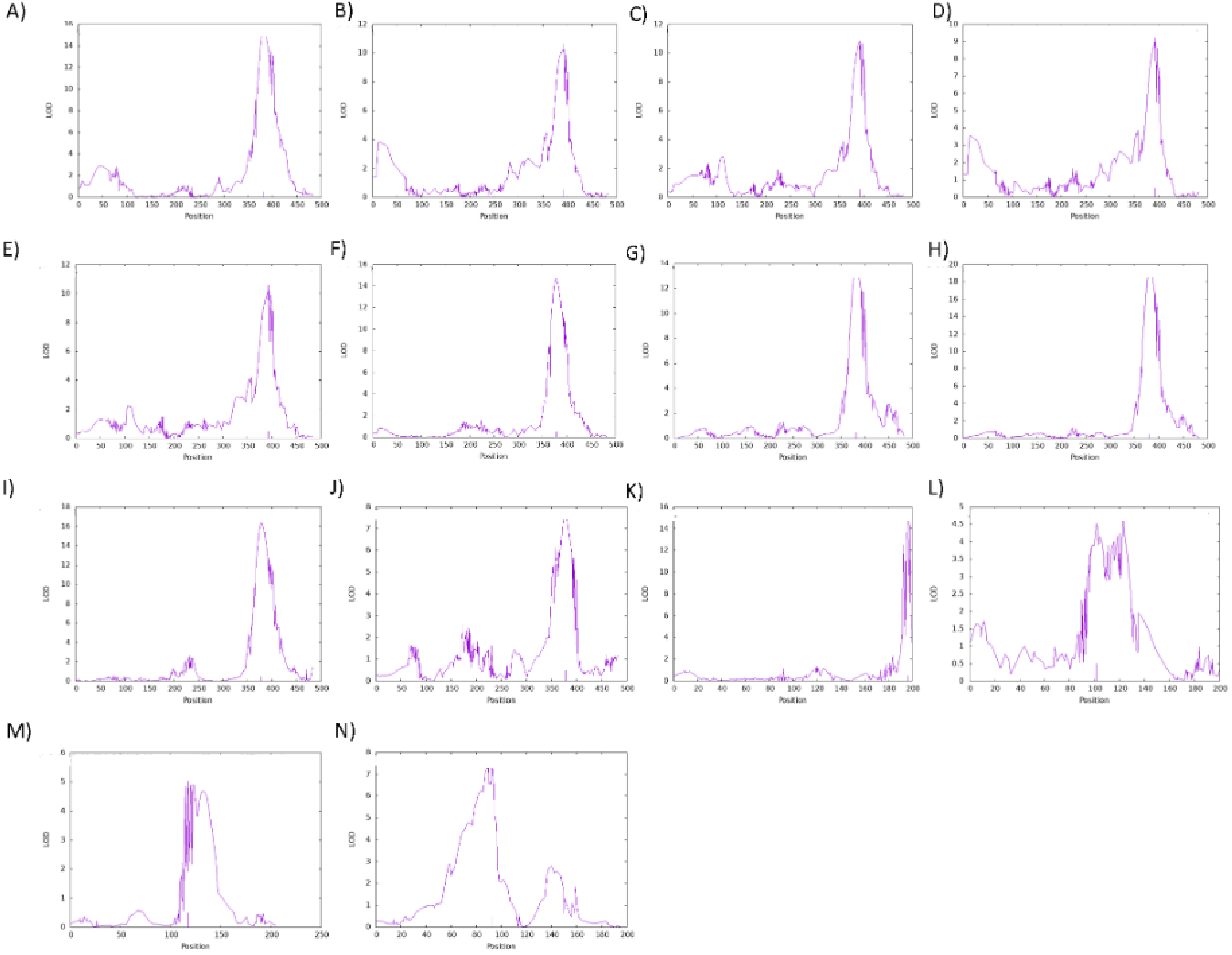
A)
B)
C)
D)
E)
F)
G)
H)
I)
J)
K)
L)
M)
N)
LOD
Position

Figure S4. Likelihood Odd Ratio plots obtained by composite interval mapping (CIM) and iterative QTL mapping (iQTL) of the frost tolerance QTLs identified in this study. A) and K) QTLs of frost damage after the first frost event of the 2016-2017 cropping season in Bretenière, (LGI and LGIII, respectively). B) Plot of the QTL involved in a reduced frost damage after the second frost event of the 2016-2017 cropping season in Bretenière, identified in LGI. C) QTL of frost damage after the third frost event of the 2016-2017 cropping season in Bretenière identified in LGI. D) QTL of area under the symptoms progress curve for frost damage after the frost period of the 2016-2017 cropping season in Bretenière, identified in LGI. E) QTL of percentage of survival after the frost period of the 2016-2017 cropping season in Bretenière, identified in LGI. F), L), M) and N) plots of the QTLs of frost damage after the first frost event of the 2017-2018 cropping season in Bretenière, identified in LGI, LGIII, LGIV and LGV (respectively). G) QTL of frost damage after the second frost event of the 2017-2018 cropping season in Bretenière, identified in LGI. H) QTL of area under the symptoms progress curve for frost damage after the frost period of the 2017-2018 cropping season in Bretenière, identified in LGI. I) QTL of percentage of survival after the frost period of the 2017-2018 cropping season in Bretenière, identified in LGI.
